# Plasma IL-23 and IL-5 as surrogate markers of lesion metabolic activity in patients with hepatic alveolar echinococcosis

**DOI:** 10.1038/s41598-018-20301-8

**Published:** 2018-03-13

**Authors:** Tuerhongjiang Tuxun, Shadike Apaer, Hai-Zhang Ma, Jin-Ming Zhao, Ren-Yong Lin, Tuerganaili Aji, Ying-Mei Shao, Hao Wen

**Affiliations:** 1 State Key Laboratory of Pathogenesis, Prevention, Treatment of High Incidence Diseases in Central Asia, First Affiliated Hospital of Xinjiang Medical University, Urumqi, China; 2 Department of Liver and Laparoscopic Surgery, Center of Digestive & Vascular Surgery, First Affiliated Hospital of Xinjiang Medical University, Urumqi, China; 3 WHO Collaborating Center for Prevention and Care Management of Echinococcosis, First Affiliated Hospital of Xinjiang Medical University and Xinjiang Centers for Disease Control, Urumqi, China

## Abstract

Fluorodeoxyglucose (FDG) uptake by alveolar echinococcosis (AE) liver lesions is a signal of their metabolic activity and of disease progression. In order to find a surrogate marker for this status, we investigated whether parameters of the peripheral and/or periparasitic immune responses were associated with metabolic activity in a prospective case-control study of 30 AE patients and 22 healthy controls. Levels of 18 cytokines and chemokines, representative of innate and adaptive immune responses, were assessed in plasma and peripheral cells of two groups of patients with (MAAE) and without (MIAE) metabolically active lesions, and in the liver of MAAE patients. Mixed cytokine profile was observed in the peripheral blood of AE patients, with a predominance of Th2, Th17 and Treg responses. Among the detected markers only plasma IL-5 and IL-23, more elevated in MAAE patients, were found discriminant. Discrimination between MAAE and MIAE patients obtained by using IL-23 was improved when IL-5 was used in combination. The combination of elevated levels of IL-5 and IL-23 is significantly associated with FDG uptake at PET scan. It offers a new tool for the follow-up of AE patients which could substitute to FDG-PET whenever non-available to assess disease progression.

## Introduction

Human alveolar echinococcosis (AE) caused by the larval stage of *Echinococcus multilocularis* (*E*. *multilocularis*) is a public health threat because of its cancer-like progression. Highest prevalence is in Northwest China, Central Asia, Western Europe, and Middle East^1,2^. Chinese health institutions have made sustained efforts to control AE since China harbors more than 90% of world’s AE burden. *E*. *multilocularis* is present in the liver in more than 95% AE patients. It invades neighbor organs and tissues, and sometimes metastasizes to distant organs including lung and brain^3^.

Therapeutic option should be highly individualized^4^. Surgery is strongly recommended when the lesion is radically removed, which occurs in less than 30% of patients^5^. Albendazole administration is a necessary complementary or single anti-infective treatment^6^. Disease progression is one of the most important parameter that greatly influences the clinical decision-making process at diagnosis and during follow-up^7^. Fluorodeoxyglucose Positron Emission Tomography (FDG-PET) is recommended to evaluate disease progression; however, the high cost and low access to this technique still bar the majority of patients from its benefits^8^.

Growing evidence supports that increased FDG uptake measured by PET in patients with progressing AE is based on the periparasitic cell infiltration and thus reflects the cross-talk between the parasite and human host’s immune cells^9^. Several studies have focused on this cross-talk and the possible role of dendritic cells, T helper (Th) cells and the cytokines they produce both in humans and experimental models^10,11^. They have usually separately addressed peripheral or regional immune response; in addition, comparison between progression, stability or regression of lesions, whenever studied, was assessed on clinical grounds only^12–14^. Comprehensive simultaneous analysis of both peripheral and local, hepatic, immune response is lacking, as well as comparison between patients with metabolically active alveolar echinococcosis (MAAE) and metabolically inactive alveolar echinococcosis (MIAE) lesions, as assessed by FDG-PET scan. Finding a surrogate marker of this metabolic activity is crucial for a proper care management strategy in any individual patient^8^. For this purpose, and in order to analyze T cell and related cytokine immune response profile in peripheral blood mononuclear cells and plasma of both MAAE and MIAE patients, as well as in the hepatic lesions of MAAE patients, this study included 30 AE patients and 22 healthy controls prospectively.

## Results

### Basic characteristics of patients

Detailed baseline information for all subjects is summarized in Table 1. Comparison of demographic characteristics between total alveolar echinococcosis patients and healthy controls (tAE *vs*. HC) as well as between subgroups (MAAE *vs*. MIAE) indicated no significant differences. Thus, all results are presented as grouped analysis.

**Table 1:** Baseline Information, Lesion Characteristics and Labs Test Results of Study Participants.

Characters\Group		tAE (n = 30)	MIAE (n = 15)	MAAE (n = 15)	HC (n = 22)	*p*
Age, years		39.5 [32.0–45.0]	37.0 [32.0–43.0]	41.0 [33.0–49.0]	33.5 [29.0–46.3]	
Sex, Male: Female		11:19	7:8	4:11	10:12	
Lesion	Diameter, in cm	88.87 [52.82–146.53]	110.2 [71.52–147.33]	76.4 [35.42–140.82]		
	Site, Right:Left	23:7	12:3	11:4	—	
Stage, No.						
	Stage I	0	0	0	—	
	Stage II	5	2	3	—	
	Stage IIIa	5	1	4	—	
	Stage IIIb	5	3	2	—	
	Stage IV	15	7	8	—	
Liver surgery, No.		15	0	15	0	
Albendazole Treatment, No		15	15	0	0	
Duration of Treatment, Month		12 [1–72]	12 [1–72]	—	—	
Blood Routine Test						
	WBC	7.60 [6.22–9.60]	8.39 [6.75–9.50]	7.31 [4.96–9.62]	6.15 [5.15–7.55]	**0**.**03**
	EO%	5.45 [3.28–8.73]	5.70 [2.30–8.70]	5.10 [3.40–10.10]	1.65 [1.05–2.68]	**0**.**00**
	BA%	0.40 [0.30–0.70]	0.40 [0.30–0.70]	0.40 [0.30–0.70]	0.50 [0.20–0.60]	0.90
	RBC	4.26 [4.02–4.67]	4.26 [4.05–4.69]	4.25 [3.95–4.63]	4.50 [4.29–4.80]	0.08
	PLT	277 [198–324]	277 [202–346]	247 [170–316]	240 [199–313]	0.61
	EO#	0.36 [0.22–0.54]	0.41 [0.12–0.55]	0.33 [0.25–0.42]	0.10 [0.07–0.21]	**0**.**00**
	BA#	0.03 [0.01–0.05]	0.03 [0.01–0.06]	0.02 [0.01–0.05]	0.03 [0.02–0.04]	0.73
Liver Function						
	CBIL	4.40 [2.50–20.60]	5.68 [3.52–32.51]	3.52 [2.44–6.49]	3.38 [2.25–4.73]	0.07
	NCBIL	7.92 [5.58–11.83]	8.14 [6.07–15.39]	7.70 [5.50–9.10]	6.74 [4.05–11.36]	0.35
	TBIL	11.20 [8.28–31.78]	14.80 [7.60–47.90]	10.60 [8.50–15.60]	10.65 [7.85–14.53]	0.24
	ALB	38.25 [35–39.70]	38.30 [35.70–39.60]	38.20 [32.80–40]	42.25 [39.93–46.8]	**0**.**00**
	ALT	20.25 [12.25–85.95]	17.6 [14.67–75.90]	24.03 [11.08–101.50]	15.75 [12.62–22.30]	**0**.**05**
	AST	23.95 [17.18–70.95]	23.90 [16.40–64.20]	24 [20.50–79.80]	17.10 [12.53–20.58]	**0**.**00**
	Γ-GT	59 [27.50–178.23]	60 [28.50–220.00]	58 [26–164.30]	14.65 [12.50–21.33]	**0**.**00**

### *Echinococcus multilocularis* Specific Serology Test

**Figure 1:**
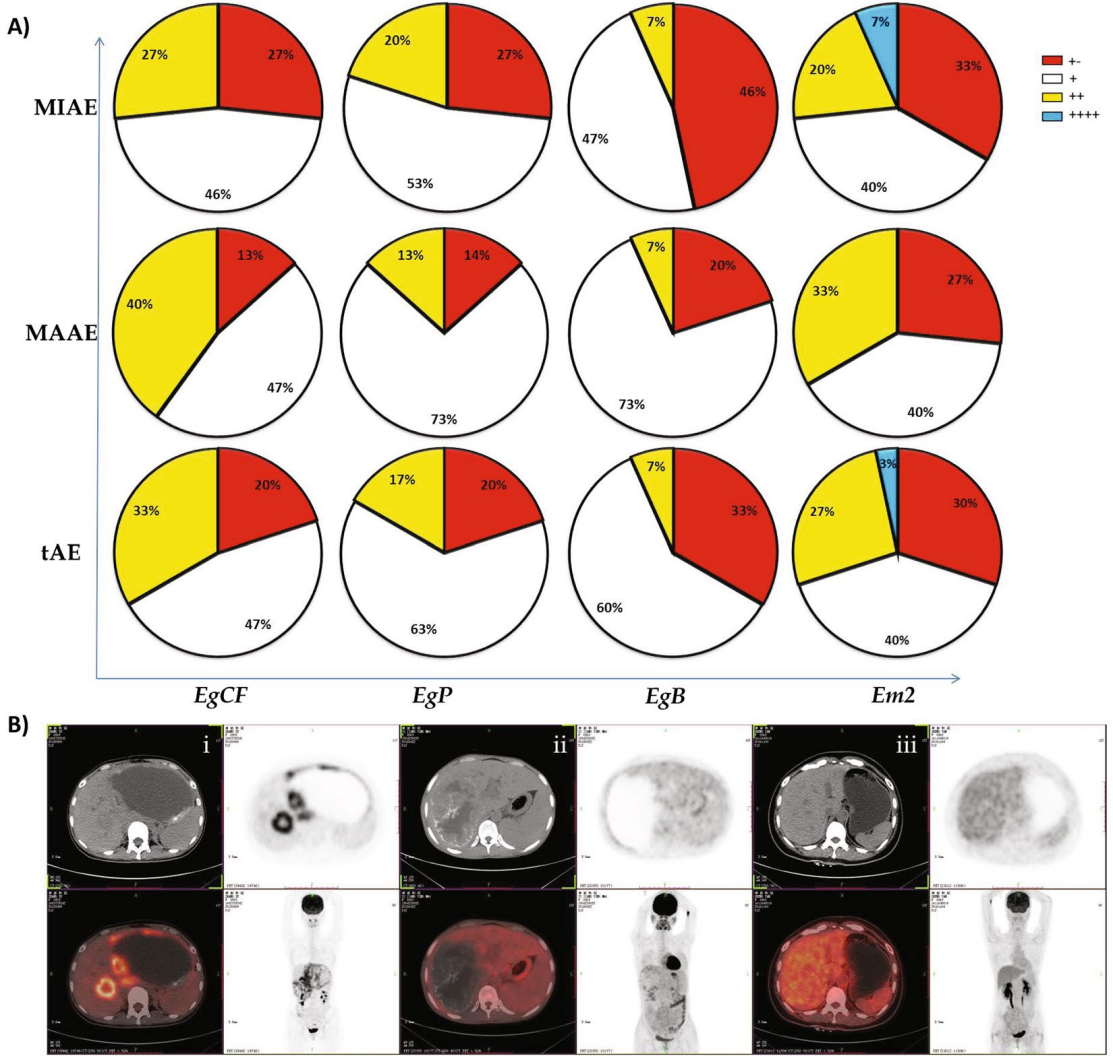
(**A)** *E*. *multilocularis* specific serology test results from AE patients. Percentage of antigen *Em2*-positive patients in MAAE was significantly higher than in MIAE patients. (**B)** 18F-fluorodeoxyglucose (FDG) positron emission tomography combined with computer tomography imaging in, MAAE (i), MIAE patients (ii) and healthy controls (iii).

Overall proportion of *Echinococcus multilocularis* specific antigen positive was significantly higher in MAAE patients than in MIAE patients. For *EgCF* antigen, there is 87% of patients with “+” to “++” in MAAE when it is 73% with MIAE group. Patients who were positive to both *EgP* and *EgB* antigen in MAAE group accounts for 86% and 80% respectively while 73% and 54% of patients were subjected in MIAE. Similar trends were also found for *Em2* antigen as shown in Fig. 1A.

### Blood counts and hepatic tests

Compared to those observed in the HC group, white blood cell (WBC), percentage and counts of eosinophil (EO%, EO#) levels were significantly higher in tAE patients. For liver functional tests, alanine aminotransferase (AST) and gamma-glutamyl transferase (Γ-GT) were also elevated significantly comparing to those in HC, while albumin (ALB) levels were lower in tAE patients (Table 1).

### Immune parameters in the peripheral blood

#### Plasma cytokines and chemokines

Compared with those observed in the HC group, the plasma levels of Th1-type cytokines interleukin (IL)-12, Th17-type cytokines IL-17A, IL-17F and IL-23, and of the regulatory cytokine IL-10 were significantly elevated in the plasma of tAE patients. IL-27 concentrations in HC group were higher than in tAE patients. When comparing with MIAE patients, concentrations of Th2-type cytokine IL-5, IL-23 were significantly higher in MAAE patients. Pro-inflammatory cytokine IL-6, IL-27 concentrations and interferon-γ (IFN-γ)/IL-5 ratio in MIAE patients were much higher than those in MAAE patients. IL-21 and IL-22 concentrations were undetectable in groups **(**Table 2**)**.

**Table 2:** Plasma Concentrations of Cytokines and Chemokines of Study Participants in Different Groups.

Item\Group	tAE (n = 30)	MIAE (n = 15)	MAAE (n = 15)	HC (n = 22)	*p* (*for MIAE *vs*. MAAE; **for tAE *vs*. HC)
**Cytokines**					
IFN-γ	297.7 [260.1–353.1]	301.1 [272.0–368.8]	297.4 [256.0–352.6]	287.7 [240.6–414.9]	
IL-5	41.43 [38.62–49.13]	40.63 [38.57–41.56]	45.06 [41.30–53.12]	39.21 [36.23–42.23]	**<0**.05*
IL-6	84.64 [20.88–148.00]	147.9 [41.0–190.9]	49.23 [13.12–122.1]	55.57 [33.67–117.3]	**<0**.01*
IL-10	7.48 [2.61–10.81]	8.41 [3.36–9.16]	7.43 [0.09–13.84]	5.33 [0–6.44]	**<0**.05**
IL-12	61.98 [45.73–74.04]	61.98 [41.85–73.92]	67.81 [47.02–74.39]	29.36 [10.96–63.19]	**<0**.05**
IL-17A	2.80 [2.04–3.35]	2.81 [2.05–3.61]	2.77 [2.03–3.35]	1.56 [1.27–2.03]	**<0**.001**
IL-17F	11.73 [9.99–15.51]	10.46 [6.10–15.02]	13.79 [10.32–16.12]	6.89 [0.75–14.76]	**<0**.05**
IL-23	96.55 [76.05–110.5]	90.06 [50.24–96.76]	105.00 [87.74–120.20]	42.67 [36.54–59.80]	**<0**.001*, **<0**.001**
IL-27	0 [0–21.67]	7.97 [0–28.93]	0 [0–0]	37.82 [20.79–48.63]	<**0**.05*, **<0**.001**
IFN-γ/IL-5	7.14 [5.79–8.58]	7.62 [6.84–9.08]	6.15 [5.60–7.76]	7.48 [6.55–8.32]	<**0**.05*
IL-10/IL-17A	2.76 [1.23–4.07]	2.98 [2.09–3.90]	2.23 [0.03–4.36]	2.21 [0–3.75]	
**Chemokines**					
CXCL1	12.05 [8.68–14.82]	11.53 [7.73–13.32]	12.69 [9.87–15.70]	13.21 [9.54–16.53]	
CXCL8	37.32 [22.62–67.94]	35.59 [11.91–126.60]	40.91 [26.04–56.06]	107.80 [54.32–251.80]	<**0**.01**
CXCL10	13.03 [6.69–19.35]	8.32 [5.84–17.54]	16.18 [10.17–22.79]	5.97 [4.57–11.06]	<**0**.05**
CXCL12α	807.3 [629.5–958.9]	703.4 [609.6–958.7]	885.5 [650.9–994.3]	695.1 [610.9–877.4]	
CCL2	191.6 [119.8–268.3]	179.8 [94.33–253.6]	236.7 [131.7–331.1]	179.3 [145.5–227.7]	
CCL3	28.10 [20.56–35.40]	25.95 [20.21–34.81]	29.40 [21.52–37.16]	45.33 [34.20–57.72]	<**0**.05**
CCL4	324.4 [260.8–388.0]	328.8 [260.9–387.8]	320.0 [260.5–452.4]	499.4 [257.4–636.8]	<**0**.05**
RANTES	85.93 [68.30–94.26]	76.10 [59.36–90.75]	92.89 [71.42–97.51]	53.41 [44.24–64.86]	<**0**.001**
Eotaxin	18.85 [11.36–26.42]	14.44 [9.98–24.36]	20.13 [17.08–32.40]	12.67 [7.75–19.84]	

Plasma chemokine levels were either similar (for CCL2, RANTES, CXCL1, CXCL10, and CXCL12α) or significantly lower (for CCL3, CCL4 and CXCL8) in tAE patients compared to HC subjects.

### Expression of the mRNA of toll-like receptor (TLRs) and of the transcription factors related to Th1- and Th2-type immune profiles in peripheral blood mononuclear cells (PBMCs)

**Figure 2:**
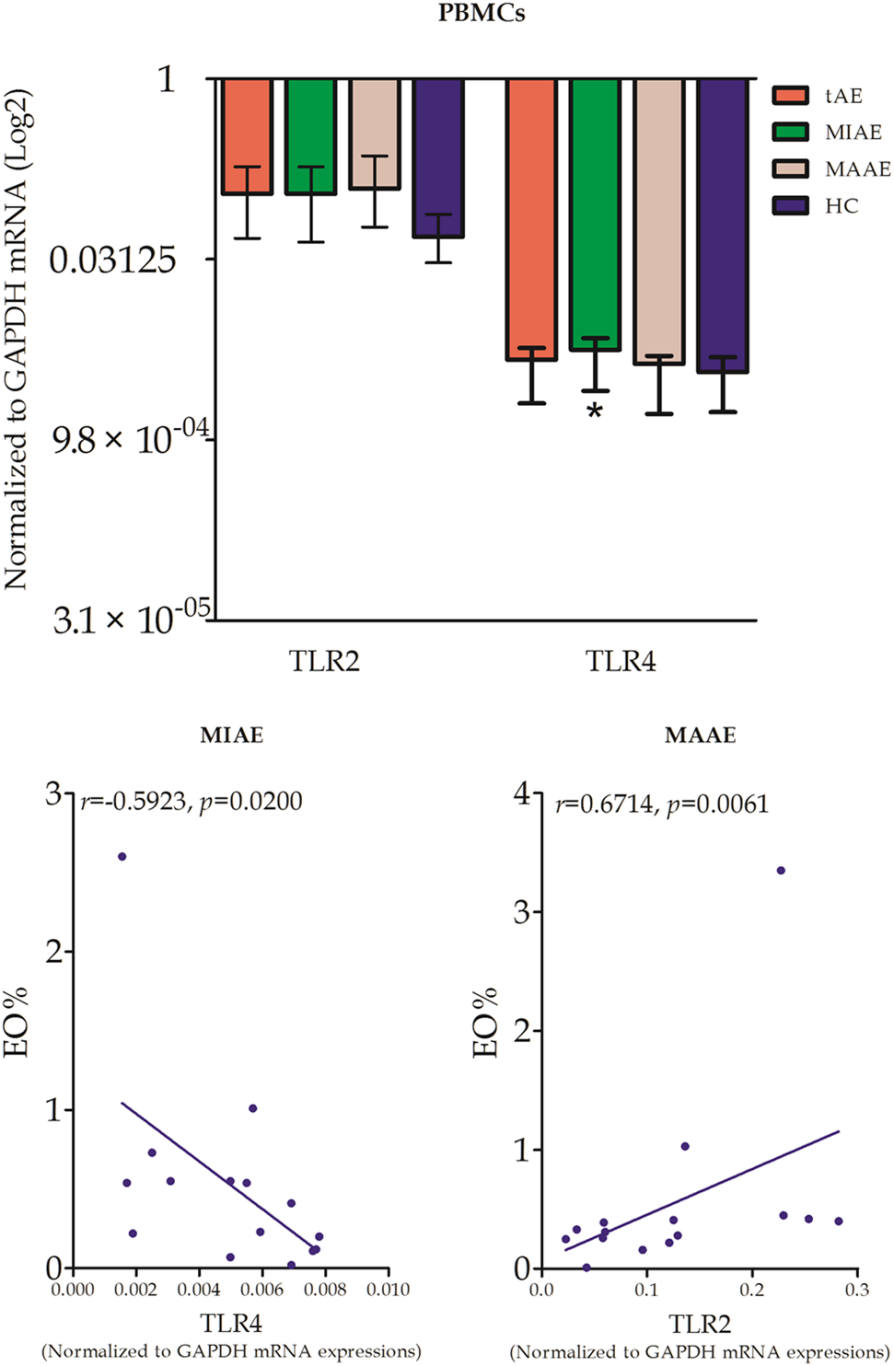
TLR2, 4 mRNA expressions and their correlations with EO% in PBMCs. Relative mRNA expressions of TLR2/4 from PBMCs in tAE patients and HC subjects were measured quantitatively. Correlation analysis between TLR2, TLR4 relative mRNA expressions and EO% levels in tAE patients were performed.

TLR4 mRNA expressions in PBMCs of MIAE patients were significantly higher than those in MAAE patients. However there was no significant difference in TLR2 mRNA levels between the two groups. We also found a positive correlation of TLR2 mRNA expressions with EO% in the blood of MAAE patients, and a negative correlation between TLR4 mRNA and EO% in the blood of MIAE patients (Fig. 2).

**Figure 3:**
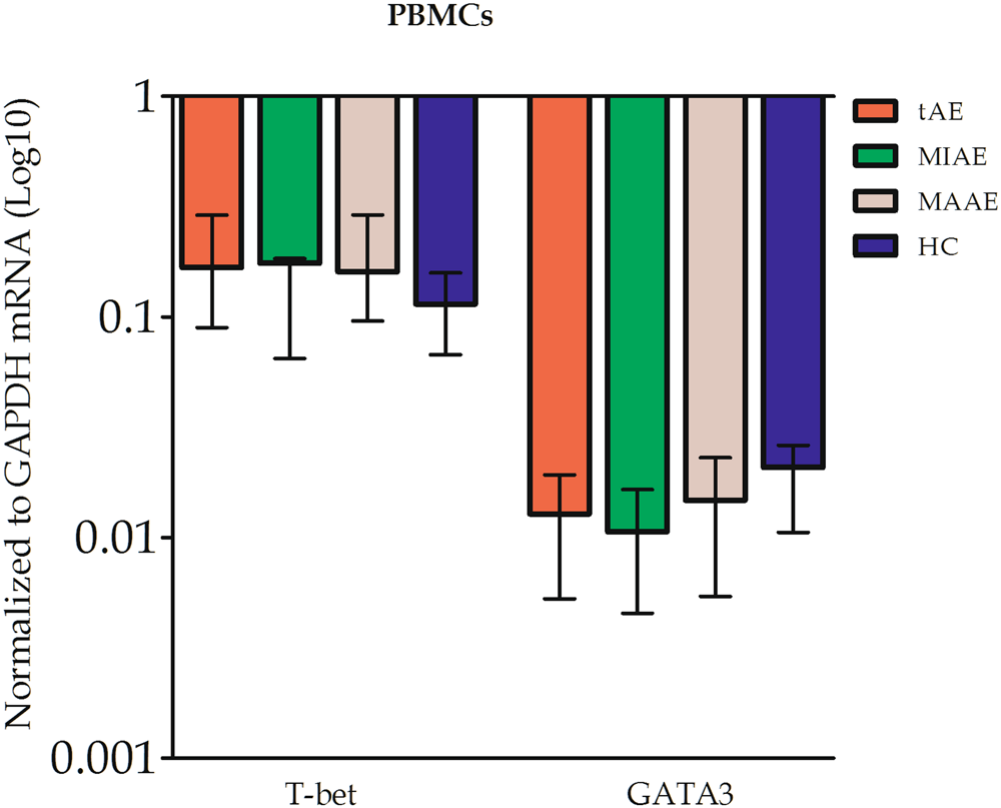
Th-1 and Th-2 cell-related transcription factors T-bet and GATA3 in PBMCs of tAE patients and HC subjects were measured.

Expression of T-bet mRNA, related to the Th1 cell profile, and GATA3 mRNA, related to the Th2 cell profile, albeit elevated in the PBMCs of tAE patients, was not significantly different from that observed in HC subjects. There was no significant difference between the expression of T-bet and GATA3 mRNA in MAAE and MIAE patients (Fig. 3).

### Immune parameters in the liver of MAAE patients

All parameters were measured in the 15 patients from the MAAE group.

### Liver expression of TLR2 and TLR4 and of transcription factors related to Th1 and Th2 immune profiles

**Figure 4:**
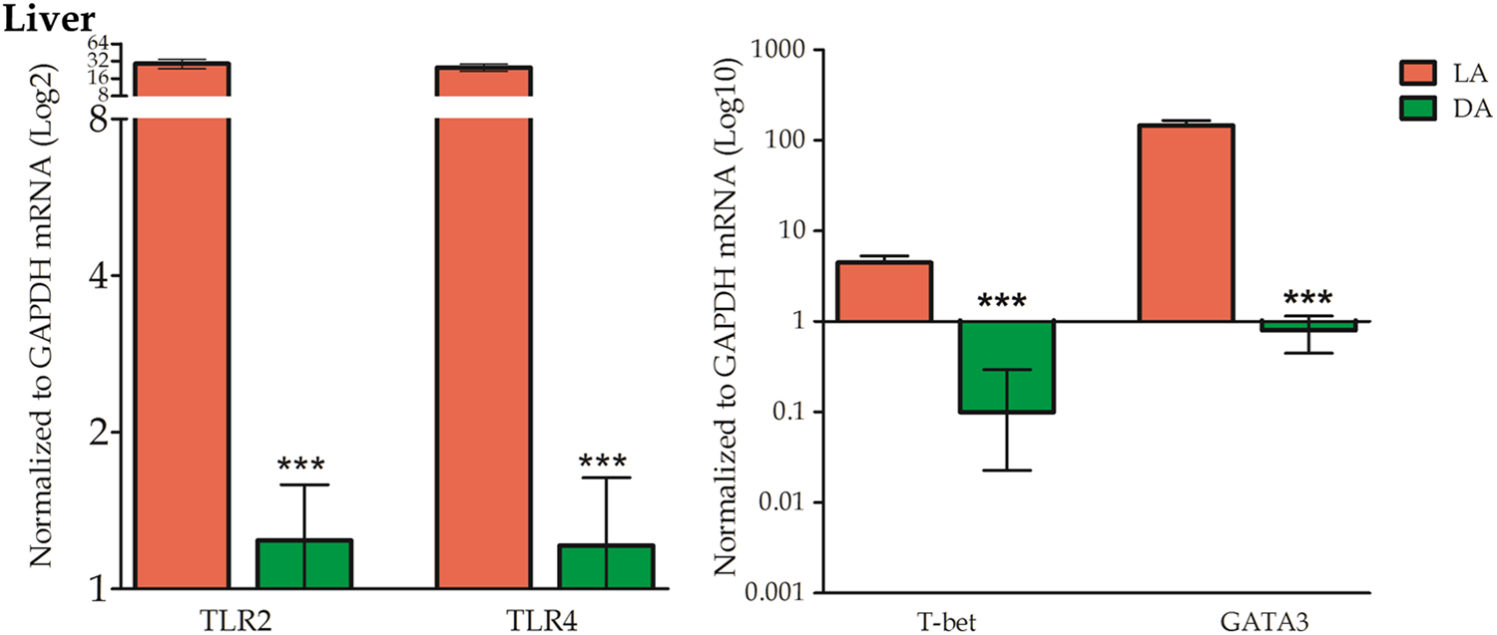
TLR2, 4 and transcription factors T-bet, GATA3 mRNA expressions in liver tissues from MAAE patients. Significantly higher TLR2, 4 and T-bet, GATA3 mRNA levels from LA liver tissues were detected comparing to DA tissues.

The mRNA expression of both TLR2 and TLR4 was very high in the AE lesions; it was significantly higher in liver tissues from lesion area (LA) than those in distant area from lesion (DA). The mRNA expression of both T-bet, associated with the Th1 profile, and of GATA3, associated with the Th2 profile, was significantly elevated in LA compared to DA. In LA, mRNA expression of GATA3 was significantly more elevated than T-bet mRNA (Fig. 4).

### Percentage of Th17 and Treg cells, and expression of Th17 -associated cytokines and transcription factors in the PBMCs

**Figure 5:**
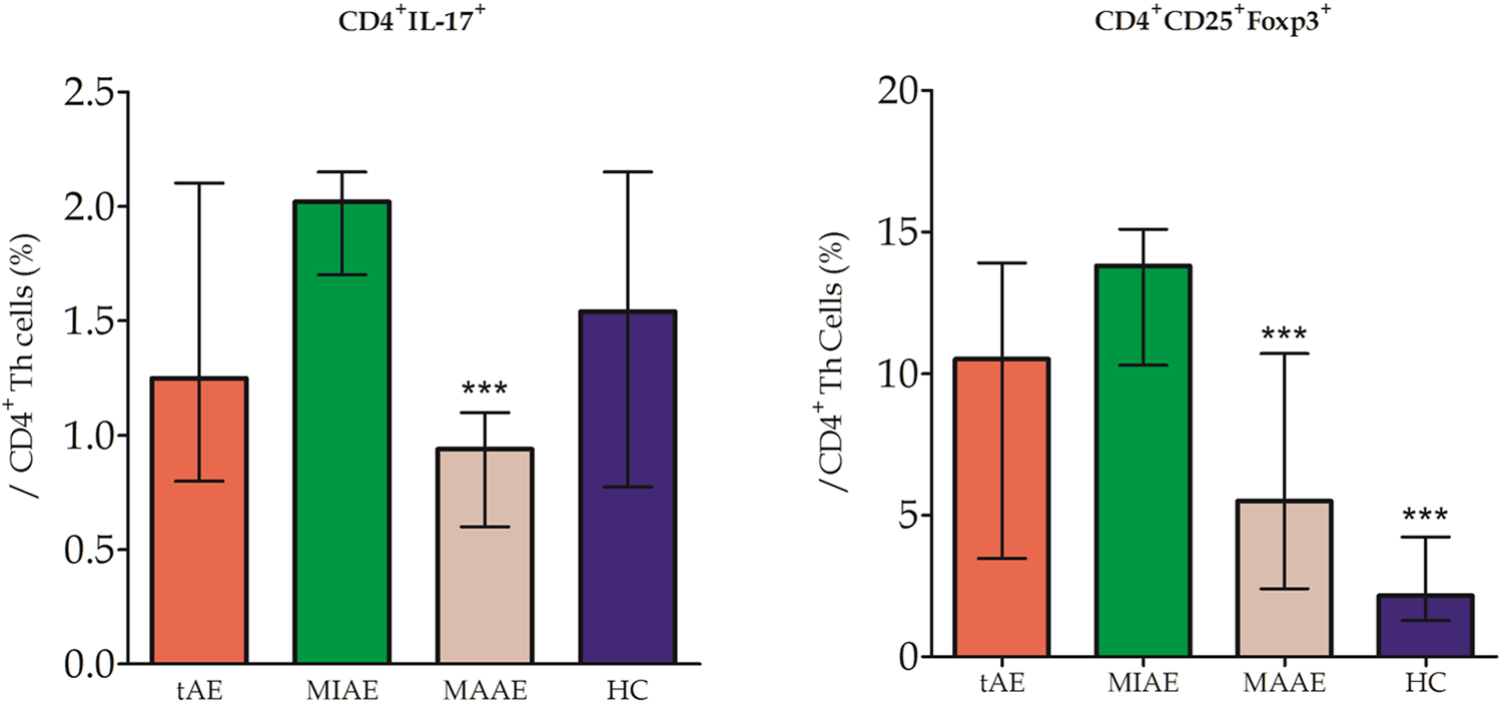
Percentage of CD4^+^ IL-17^+^ Th17 and CD4^+^ CD25^+^ FoxP3^+^ Treg cells in PBMCs. Median percentage levels of Th17 cells in MAAE patients were higher than that in MIAE patients. The percentage of Treg cells was very elevated in tAE and MIAE patients comparing to HC and MAAE subjects.

Median percentage of peripheral CD4^+^ IL-17^+^ Th17 cells in tAE patients was similar to that in HC subjects; When comparing with MAAE patients, significant high proportion of CD4^+^ IL-17^+^ Th17 cells was found in MIAE patients. The percentage of CD4^+^ CD25^+^ FoxP3^+^ Treg cells was very elevated in tAE patients, significantly higher than in HC subjects; It’s proportion in MAAE patients was lower than that in MIAE patients (Fig. 5).

**Figure 6:**
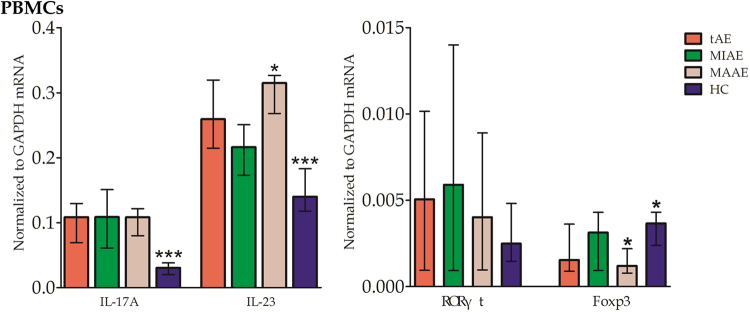
Th17, Treg -associated cytokines IL-17A, IL-23 and transcription factors RORγτ, FoxP3 mRNA levels in PBMCs. Increased IL-17A and IL-23 expressions were found in tAE patients comparing with HC. IL-23 levels were elevated in MAAE than in MIAE patients. FoxP3 mRNA expressions were significantly lower in the tAE and MAAE patients when comparing to those in HC and MIAE.

Significantly elevated expression of IL-17A and IL-23 mRNAs in the PBMCs were found in tAE patients compared with HC subjects. IL-23 expressions in MAAE patients were significantly higher than those in MIAE patients. The expression levels of FoxP3 mRNA were higher in HC subjects and MIAE patients than those in tAE and MAAE patients (Fig. 6).

### Liver expression of the Th17 and Treg associated cytokines and transcription factors

**Figure 7:**
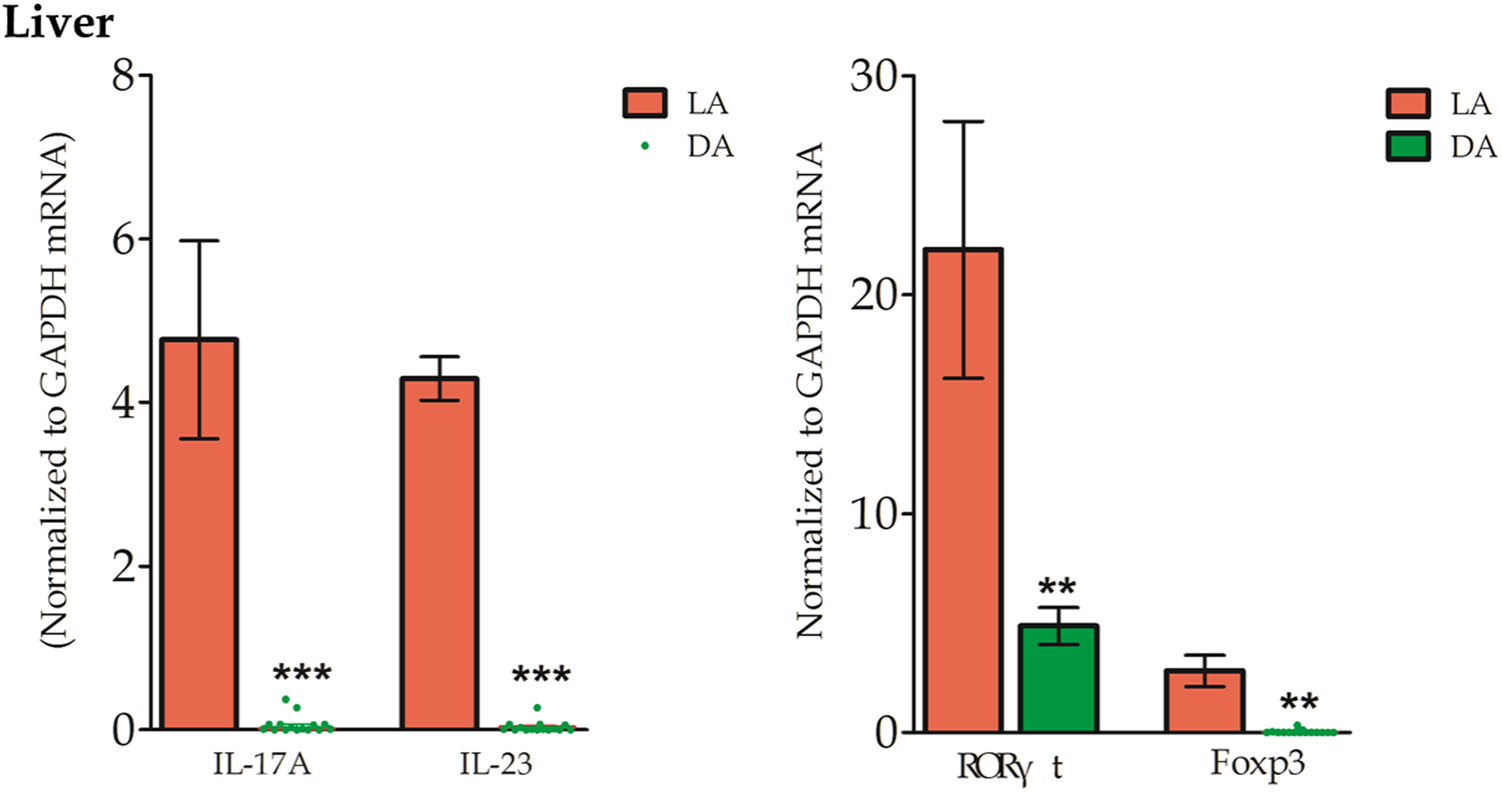
Th17, Treg -associated cytokines IL-17A, IL-23 and transcription factors RORγτ, FoxP3 mRNA levels in liver tissues. IL-17A and IL-23 mRNA were elevated in tAE and MAAE patients’ LA comparing with DA of liver tissues. Both RORγτ mRNA and FoxP3 mRNA expressions were significantly more expressed in LA.

IL-17A and IL-23, associated with the Th17 and Treg profile respectively, mRNA expressions were significantly increased in LA than that in DA. Their corresponding specific transcription factors RORγτ and FoxP3 were significantly more expressed in LA (Fig. 7).

**Figure 8:**
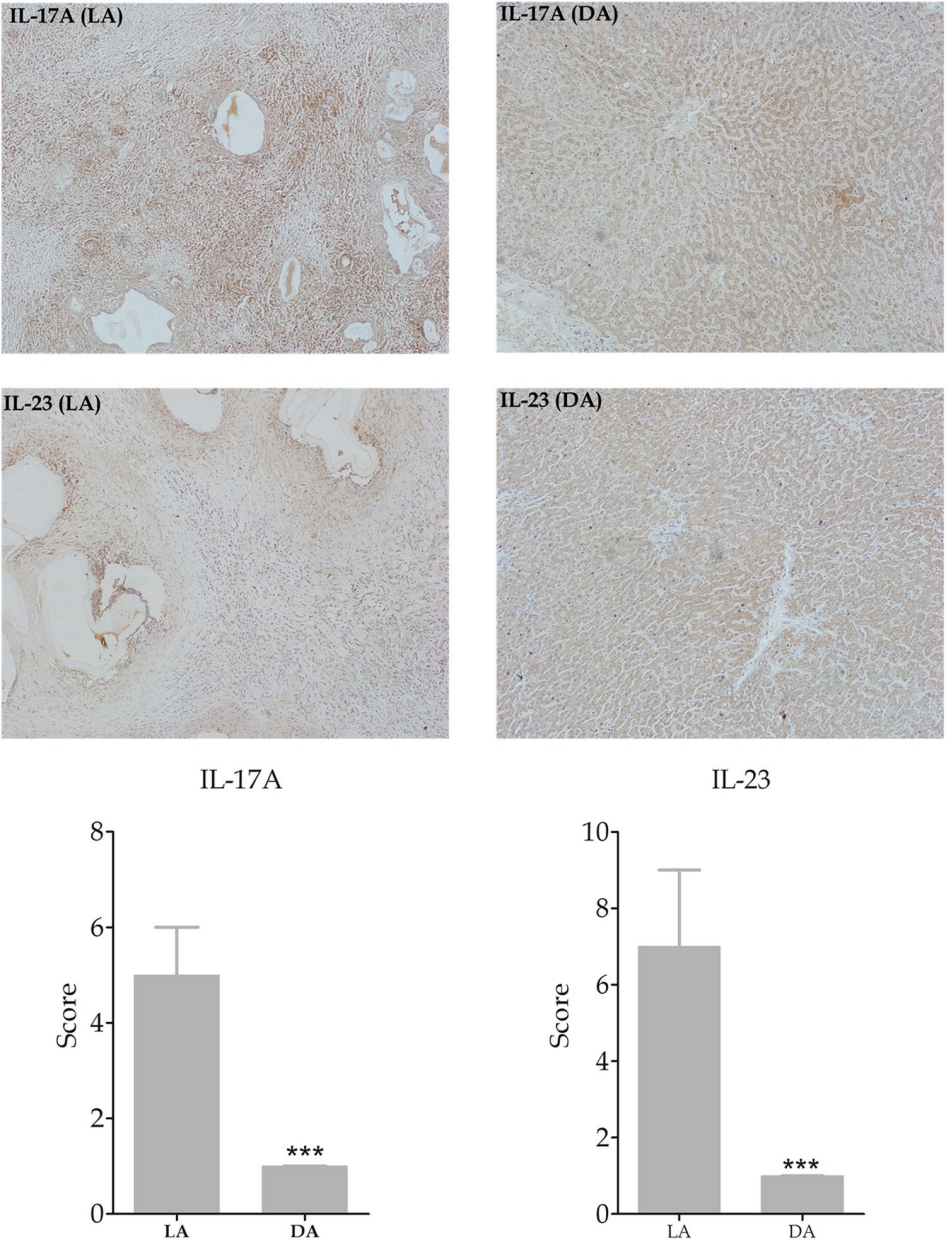
Immunohistochemical expression of IL-17A and IL-23 in LA and DA of liver tissue (Final magnification: 100X). IL-17A and IL-23 expression was significantly higher in LA than in DA of liver tissue.

In addition, results of immunostaining for IL-17A and IL-23 showed that both of them were significantly more expressed in LA than in DA as shown in Fig. 8.

### Diagnostic value of plasma levels of IL-5, IL-23 and combinations for predicting the different metabolic activities

**Figure 9:**
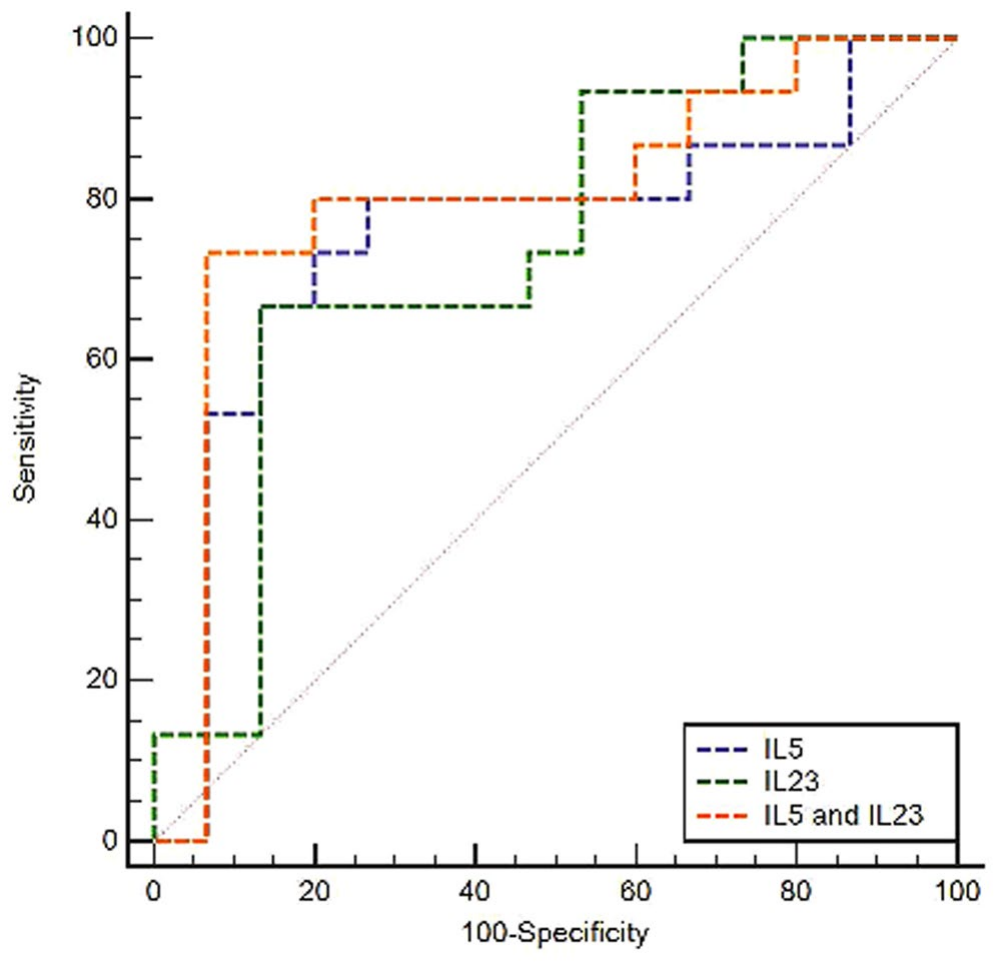
Receiver operating characteristic curves (ROCs) for plasma levels of IL-5, IL-23 and their combination to predict the metabolic activity of AE lesions.

Detailed results of ROC curves analysis are given in Fig. 9 and Supplementary Table S1. Combination of IL-5 and IL-23 was the best surrogate marker for FDG uptake-related metabolic activity with 73% sensitivity and 93% specificity, positive predictive value of 92% and negative predictive value of 78% for a cut-off point of 52.6.

## Discussion

In the liver of patients with AE, the periparasitic infiltrate by immune cells is one of the pathological hallmarks of the disease^1,4^. A set of evidence now attributes the periparasitic increased uptake of FDG detected by PET in the liver of the patients^9^ to this periparasitic immune reaction, and not to the metacestode itself^7,8^. No study had been performed to assess the relationship, if any, between the metabolically active status and peripheral parameters of the immune response that could be used as surrogate markers. Our results globally confirmed the mixed cytokine profile observed in the peripheral blood of AE patients, with a predominance of Th2, Th17 and Treg responses; however, most of the cytokines and all chemokines measured in the plasma as well as the relevant cell subpopulations and associated transcription factor mRNA expression in the PBMCs, were not discriminant to distinguish between patients with metabolically active and inactive lesions. Only plasma IL-5 and IL-23, more elevated, and IL-6 and IL-27, less elevated, in MAAE patients were found discriminant. Additionally, our results show that mRNA of TLRs as well as of transcription factors involved in the activation of Th2-, Th17- and Treg-types of response is actually highly expressed in the metabolically active lesions, which strongly supports the results obtained in peripheral blood.

Currently, FDG-PET is the only option to predict metabolic activity of the liver lesions in AE patients^15,16^, and plays a crucial role in therapeutic decisions and patient’s follow-up^4,17,18^. However, the majority of AE patients are from poorly resourced regions and cannot benefit from this technique because of the necessary specific equipment, trained specialists and the unaffordable cost. Since FDG uptake detected by PET depends on the periparasitic infiltrate by immune cells^7^, it was rational to further explore the possible immune response marker(s) associated with positive FDG-PET. In this study, IL-5 and IL-23 were the best predictors of metabolically active lesions, and can thus be regarded as potential surrogate markers; adding IL-27 and IL-6 did not add to the discrimination power. Such cytokine measurements in plasma are non-invasive, compared to PET, and might be available and affordable in most settings even in poor resourced regions around the world, at least in reference centres. Since the early reports on the cytokine profile in AE patients^19,20^, highly increased IL-5 production by the PBMCs has been constantly found. Major involvement of Th2 cells and related cytokines in the immunopathogenesis of AE is fully confirmed by the expression of their transcription factors in the liver of the MAAE patients included in this study. Our study is, however, the first to show that different levels of IL-5 are found depending on the metabolic activity of AE lesions. The low levels of IL-27 in MAAE patients also support the relative failure of the Th1 type of response already reported in previous studies^12,21^. In addition, we showed the elevation of IL-23 in AE patients, and its capacity to discriminate between metabolically active and inactive lesions. TLR2 and TLR4 are known to regulate T cell differentiation in infectious diseases^22–24^. The elevated TLR2 and TLR4 expression we found in AE patients might help induce IL-23 production; the association we observed with circulating eosinophil, highly influenced by IL-5 levels, suggests TLR involvement in the differentiation of the Th2 profile. IL-23 is an important signal mediator of Th17 cell functional differentiation^25,26^. It also serves to stabilize and expand Th17 cell functions and to enhance the expression of IL-17 in granulomatous lesions^27,28^. The immunopathological consequences of increased IL-17 production have been shown in both human and experimental AE^10,11^. Besides, elevated Th17 cell response might increase angiogenesis and inflammation that provides a new environment and nutrition to the parasite while accelerating disease progression^29^. It is acknowledged, however, that whether Th17 become pathogenic or not can be taken independently of IL-23^30^; on the other hand, the modifications of the various isotypes of IL-17 we found are not totally similar to those reported in a previous study on human AE^13^; this might be due to the variety of disease stage, as was observed in experimental mice^11^. Paradoxically, we found a lower percentage of Th17 as well as Treg cells and associated transcription factors in the PBMCs of MAAE. This could be explained by the high level of secretion of IL-23 locally and the ‘homing’ of Th17 and Treg cells to the lesions, thus resulting in relative decrease at the periphery. Such an observation was made for the low percentage of CD8^+^ T cells and high CD4^+^/CD8^+^ T cell ratio in the PBMCs of AE patients, which contrasted with the high percentage of CD8^+^ T cells in the lesions^31^. The low levels of IL-6, a key-proinflammatory cytokine, in MAAE patients, might also look paradoxical. However, IL-6 is a potent inducer of acute phase proteins, and the absence of elevation of C-reactive protein in patients with otherwise advanced lesions of AE has been mentioned since the very beginning of clinical immunology studies of AE^32^; mechanisms for this IL-6 down-regulation are still unknown.

Study limitations include the absence of data on the local response in the liver of MIAE patients, since only patients from the MAAE group underwent surgery. The immune profile of the patients might have been modified by the various durations of albendazole administration. However, it is the first comparison of the peripheral immune response in patients with different metabolic activities in their liver lesions, as assessed by FDG uptake, and which studied relevant parameters of the immune response both at the periphery and in AE liver lesions with proven metabolic activity. We could not provide evidence that IL-10 levels, strikingly elevated in AE patients^14^, were discriminant between metabolically active/inactive patients. From preliminary studies, we did not include transforming growth factor (TGF)-beta among the cytokines measured for this study. However, because of its role and that of IL-23 in the tolerance process and the subtle balance between Th17 and Treg types of immune response, TGF-beta should certainly be included in the necessary prospective studies which will be designed in the future to confirm the usefulness of the surrogate markers of AE lesion metabolic activity we describe in this study.

## Materials and Methods

### Ethical Statement

Management of the human subjects in strict compliance with Helsinki declaration, 2013^33^ was approved by the appropriate Ethical Committee of the First Affiliated Hospital of Xinjiang Medical University (Approving Number: 20120321-1). Informed consents were obtained from all participants or their legal custodies in their own languages.

### Enrollment Policy

**Figure 10:**
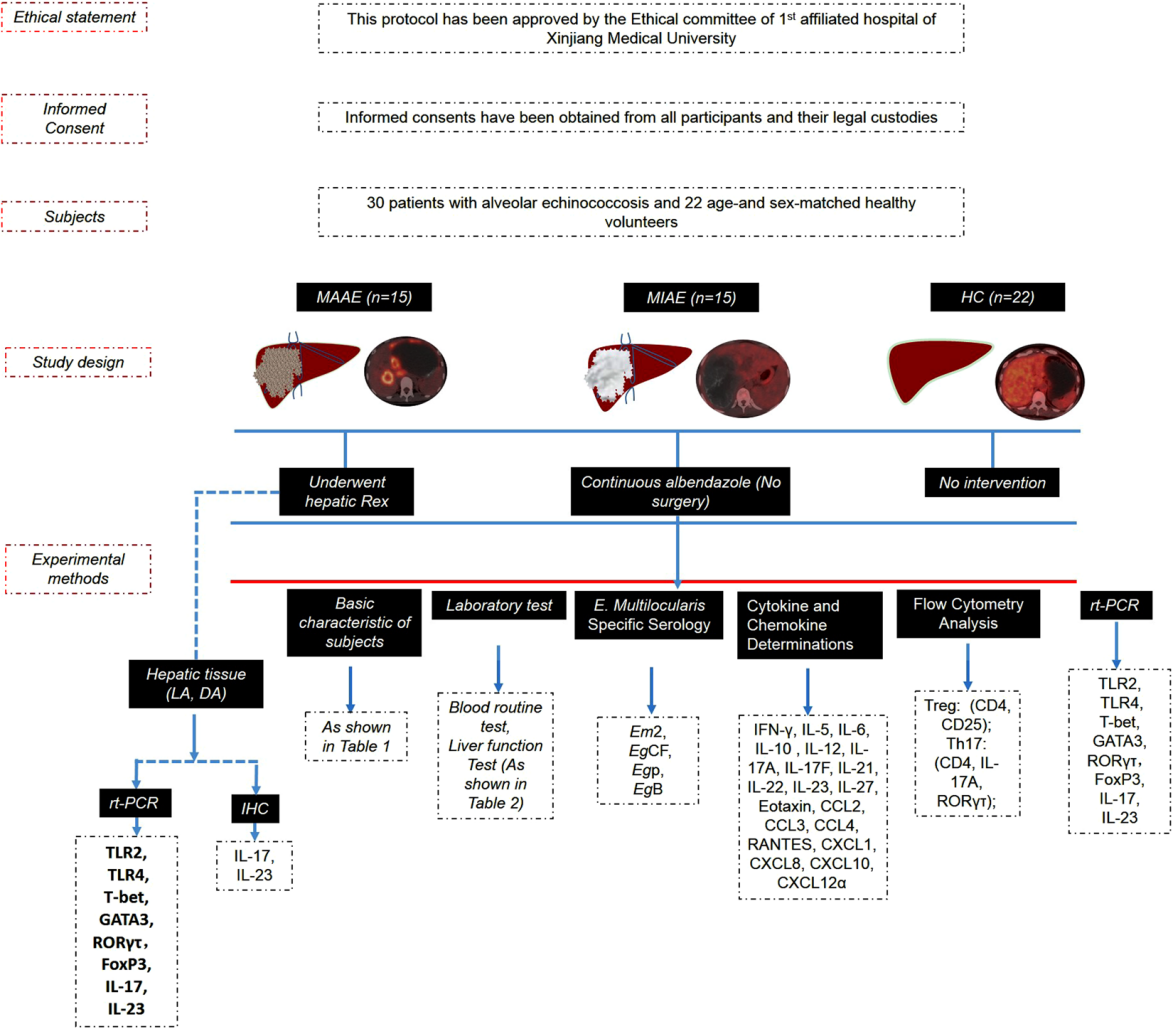
Algorithm of study design.

A prospective case-control study was conducted in 30 consecutive eligible AE patients during November 2012 to April 2015. Inclusion criteria were: 1) AE diagnosis, according to the international recommendations^34^; 2) consent of the patient for the performance of FDG-PET scan and acceptance of medical follow-up; 3) possible administration of albendazole^34^, as first intent to treat. Recruitment of patients stopped when 2 groups of 15 patients each, based on FDG-PET scan results, were available for analysis. Meanwhile, 22 age-and sex-matched healthy volunteers were recruited as healthy controls (HC). Exclusion criteria were: 1) association of acute or chronic diseases, other than AE, and pregnancy; 2) administration of any anti-inflammatory agents, immunosuppressant, and anti-parasitic drugs other than albendazole; 3) clinically expressed biliary complications and bacterial infection. The detailed algorithm of study design is shown in Fig. 10.

### Baseline Information

Detailed clinical baseline characteristics of subjects were meticulously recorded. AE patients were clinically staged according to World Health Organization Informal Working Group on *Echinococcosis* PNM classification^35^.

### Assignment to groups on the basis of lesion ‘metabolic activity’

FDG-PET scan was performed after an average of 12 month-albendazole administration. Images of FDG-PET scan were analyzed by two experienced nuclear physicians with expertise in AE, blinded to clinical, biological and pathological characteristics of the patients. The PET/CT acquisitions were performed 3 h after ^18^F-FDG injection as recommended^16^. Any patient found with metabolically active lesions was assigned to the MAAE group and operated on, and any patient found with metabolically inactive lesions was assigned to the MIAE group and continuously treated by albendazole (Fig. 1B).

### Laboratory Examination

Routine blood cell counts, including WBC, EO, percentage of basophilic (BA%), red blood cell counts (RBC) and platelets (PLT) as well as liver function tests including conjugated/non-conjugated/total bilirubin (CBIL/NCBIL/TBIL), alanine aminotransferase (ALT), were measured in all subjects using the common laboratory test procedures.

### *Echinococcus multilocularis* Specific Serology Analysis

*E*. *multilocularis* specific serology tests used the dot immunogold filtration assay technique with semi-quantitative evaluation of the results scored”+” to “++++”^36^. Briefly, serum samples from tAE patients were diluted with 20 mM Tris–HCl (pH 8.2) and completely infiltrated on the nitrocellulose paper. After washing once, samples were incubated with colloidal gold conjugated anti-human IgG antibody (Sigma I1886, USA). Protein antigens of *EgCF*, *EgP*, *EgB*, and *Em2* were added in sequence after the previous one was totally absorbed and the results were detected immediately after the last washing buffer filtrated. In general, the intensity of the red colour indicated the degree of immune combination which reflected the antibody activity levels in samples. “+” to “++++” marker was applied for the intensity of red colour when “±” was considered to be “doubtful”.

## Samples Preparation

### Blood Samples

Plasma was prepared and stored at −80 °C until use^37^. PBMCs were isolated using the Ficoll-Hypaque (Sigma-Aldrich, St Louis, MO, USA) density gradient centrifugation technique^37^.

### Liver Tissue

Liver tissue sections were obtained from the 15 MAAE patients during the surgical procedure^5^. The specimens were taken from the AE lesion area (LA) and from a macroscopically normal distant area (DA) of liver tissue.

## Flow Cytometry Analysis of Th17 and Treg cell subpopulations

### Cell preparation

For the analysis of Th17 cells, PBMCs were suspended at a density 1 × 10^6^ cells/ml in complete culture medium (RPMI 1640 supplemented with 100 U/ml penicilin and 100 ug/mL streptomycin, 2 mM glutamine and with 10% heat-inactivated fetal calf serum, Gibco, USA). The cell suspension was transferred to each well of 24-well plates. Cultures were stimulated with phorbol myristate acetate (PMA, 50 ng/mL) plus ionomycin (1 uM, all from Alexis Biochemicals, San Diago, CA), in the presence of monensin (500 ng/mL, from eBioscience, San Diago, CA) in an incubator at 37 °C under a 5% CO_2_ environment. After 5 h of culture, the contents of the well were transferred to 15-mL sterile tubes. The cells were than centrifuged at 1500 rpm for 5 min. For the analysis of Tregs, PBMCs were aliquoted into tubes for further staining^38^.

### Surface and intracellular staining

Cells were aliquoted into tubes and washed once in phosphate-buffered saline (PBS). For Th17 analysis, the cells were incubated with fluorescein isothiocyanate (FITC) anti-human CD4 monoclonal antibody (Ab) at 4 °C for 20 min. For Treg analysis, the cells were incubated with FITC anti-human CD4 and phycoerythrin (PE) anti-human CD25. After the surface staining, the cells were stained with fluorescein phycoerythrin (PE) anti-human IL-17A for Th17 detection or PC5 anti-human FoxP3 for Treg detection after fixation and permeabilization according to the manufacturer’s instructions. Isotype controls were performed to enable correct compensation and confirm Ab specificity. All of the Abs were from eBioscience, San Diego, CA. Flow cytometry analysis was performed using Flow Jo software.

### Plasma Cytokine and Chemokine Determinations

Plasma concentrations of 18 cytokines and chemokines of including IFN-γ (sensitivity is 0.2 pg/ml), IL-5 (0.3 pg/ml), IL-6 (0.4 pg/ml), IL-10 (0.1 pg/ml), IL-12 (0.04 pg/ml), IL-17A (0.1 pg/ml), IL-17F (0.1 pg/ml), IL-21 (0.6 pg/ml), IL-22 (8.2 pg/ml), IL-23 (0.9 pg/ml), IL-27 (5.1 pg/ml), Eotaxin (1.4 pg/ml), CCL2 (0.6 pg/ml), CCL3 (1.1 pg/ml), CCL4 (4.7 pg/ml), RANTES (0.2 pg/ml), CXCL1 (2.8 pg/ml), CXCL8 (1.2 pg/ml), CXCL10 (0.3 pg/ml), CXCL12α (20.5 pg/ml) were detected by using Luminex bead-based multiplex assay (eBiosciences, San Diego, CA, USA) under close compliance with manufacturer’s guidelines.

### Quantitative Real-time PCR Analysis of Toll like receptors, cytokines and transcription factors

Measurement of mRNA expression of TLRs, cytokines and transcription factors, namely TLR2 and TLR4, IL-17 and IL-23, and associated transcription factors relevant to the study, T-bet (for Th1 cells), GATA3 (for Th2 cells), RORγτ (for Th17 cells), and FoxP3 (for Treg cells), was performed using SYBR Green System on PBMCs for all AE patients and HC subjects, as well as on liver samples of patients from the MAAE group (who were operated on).

Total RNA was obtained using TRIzol (Invitrogen) according to the manufacturer’s instructions and quantified by A260 absorbance. 2 μg of RNA was reverse-transcribed to cDNA, and used as a template for Real-time PCR. qRT-PCRs were conducted with the SYBR Green PCR premix following the manufacturer’s protocols (Invitrogen, CA, USA) with primers purchased from Sangon, Shanghai (see Supplementary Table S2). Measurements were performed using SYBR Green program on i-Q 5.0 Real-time PCR system (BioRad, Foster City, CA, USA). The relative amounts of PCR products were determined using the relative standard curve method and GAPDH was used as an internal control. The 2^−ΔΔCt^ method was used to determine the specific Ct value of each target gene.

### Immunohistochemistry of IL-17A and IL-23

Liver tissue samples were prepared for immunohistochemistry, and a semi-quantitative method was applied for analyzing IL-17A and IL-23 immunostaining. Two senior pathologists blinded to each other’s results read the sections; scores considered both staining intensity and the percentage of cells stained at a specific range of intensity, as described previously^39^.

### Statistical Analysis

All continuous variables were expressed as *median* and *interquartile range* (*IQR*) and categorical variables as number and percentage. All AE patients were compared with HC subjects and MAAE with MIAE patients. In the liver, measurements performed in LA were compared with those performed in DA. We used Mann-Whitney rank-sum test to detect the differences between groups and Spearman correlation analysis to test correlation between two continuous variables. All tests were two-sided and a probability value of *p* ≤ 0.05 was considered to be statistically significant. Evaluation of the diagnostic values for IL-5, IL-23 and their combination was made using Receiver Operative Curves (ROC). Areas under curves (AUC) and sensitivity (Se), specificity (Sp), positive predictive value (PPV), and negative predictive value (NPV) were calculated and compared between groups. Statistical analysis of the data was conducted with the Statistical Package for Social Sciences (SPSS), version 17.0.

## Electronic supplementary material

Supplementary Tables
