# Supplementary material for: Plasma IL-23 and IL-5 as surrogate markers of lesion metabolic activity in patients with hepatic alveolar echinococcosis
Source: Sci Rep. 2018 Mar 13;8:4417. doi: 10.1038/s41598-018-20301-8 (PMC5849767; doi:10.1038/s41598-018-20301-8)
Supplement: Supplementary file 1 — Supplementary Tables [file 41598_2018_20301_MOESM1_ESM.pdf]

Plasma IL-23 and IL-5 as surrogate markers of lesion metabolic activity in patients with hepatic alveolar echinococcosis

Short Title: Predictive markers in progressive AE patient

Tuerhongjiang Tuxun,[1, 2, 3, #] Shadike Apaer,[1, 2, 3, #] Hai-Zhang Ma,[2, 3] Jin-Ming Zhao,[1, 2, 3] Ren-Yong Lin,[1, 3] Tuerganaili Aji,[1, 2, 3] Ying-Mei Shao,[1, 2, 3] Hao Wen*,[1, 2, 3]

Supplementary materials

Supplementary Table 1. Diagnostic Value of Different Plasma Level of IL-5, IL-23 and Their Combination for Predicting Metabolic Activity in AE Patients

	Cut-off point	AUROC (95% CI)	Sensitivity (%)	Specificity (%)	PPV (%)	NPV (%)
**IL-5**	40.6	0.756 (0.565 - 0.893)	80.0	73.3	75.0	78.6
**Il-23**	96.8	0.743 (0.551 - 0.884)	66.67	86.67	83.3	72.2
**IL-5 + IL-23**	52.632	0.800 (0.614 - 0.923)	73.33	93.33	91.7	77.8

AUROC, area under receiver operating characteristic curve; PPV, positive predictive value; NPV, negative predictive value

Supplementary Table 2. Primers Used for Real-time PCR Analysis

Gene	Forward Primers (5′-3′)	Reverse Primers (5′-3′)	ReSeq ID
TLR2	GCCAAAGTCTTGATTGATTGG	TTGAAGTTCTCCAGCTCCTG	NM_001318796.1
TLR4	TGGATACGTTTCCTTATAAG	GAAATGGAGGCACCCCTTC	NM_138557.2
T-bet	TGTTGTGGTCCAAGTTTAATCAGCA	CCCGGCCACAGTAAATGACAG	NM_013351.1
GATA3	GGACCCCATCTGTGAATAAGC	GCTCTCTGAAACCC TCAATGG	XM_005252442.2
L-17A	GGACTGTGATGGTCAACCTG	GGAGATTCCAAGGTGAGGTG	NM_002190.2
IL-23	GACACATGGATCTAAGAGAAGAG	AACTGACTGTTGTCCCTGAG	NM_016584.2
RORγτ	CCTGGGCTCCTCGCCTGACC	TCTCTCTGCCCTCAGCCTTGCC	NM_001001523.1
Foxp3	CTCAAGCACTGCCAGGCGGAC	CAGCGGATGAGCGTGGCGTAGG	XM_017029567.1
GAPDH	GCACCGTCAAGGCTGAGAAC	TGGTGAAGACGCCAGTGGA	NM_001289746.1
